# Supplementary material for: A resource to empirically establish drug exposure records directly from untargeted metabolomics data
Source: Nat Commun. 2025 Dec 9;16:10600. doi: 10.1038/s41467-025-65993-5 (PMC12689629; doi:10.1038/s41467-025-65993-5)
Supplement: Supplementary file 2 — Description of Additional Supplementary Files [file 41467_2025_65993_MOESM2_ESM.pdf]

Description of Additional Supplementary Files

Supplementary Data 1: Community-curated list of delta mass interpretations.
Supplementary Data 2: Delta masses accepted in the drug analog library. Delta masses were calculated as the precursor masses of drug analogs minus those of drugs.
Supplementary Data 3: Source of drugs used in synthetic microbial community incubation.
Supplementary Data 4: Bacterial strains used in the six synthetic microbial communities. hCom, human gut microbiota.
Supplementary Data 5: Composition of the Brain heart infusion (BHI) medium for anaerobic microbial cultures.
