# Supplementary material for: A resource to empirically establish drug exposure records directly from untargeted metabolomics data
Source: Nat Commun. 2025 Dec 9;16:10600. doi: 10.1038/s41467-025-65993-5 (PMC12689629; doi:10.1038/s41467-025-65993-5)
Supplement: Supplementary file 4 — Reporting summary [file 41467_2025_65993_MOESM4_ESM.pdf]

nature portfolio

Corresponding author(s): Pieter C. Dorrestein

Last updated by author(s): Oct 1, 2025

Reporting Summary

Nature Portfolio wishes to improve the reproducibility of the work that we publish. This form provides structure for consistency and transparency in reporting. For further information on Nature Portfolio policies, see our Editorial Policies and the Editorial Policy Checklist.

Statistics

For all statistical analyses, confirm that the following items are present in the figure legend, table legend, main text, or Methods section.

n/a	Confirmed	
☐	☒	The exact sample size (*n*) for each experimental group/condition, given as a discrete number and unit of measurement
☐	☒	A statement on whether measurements were taken from distinct samples or whether the same sample was measured repeatedly
☐	☒	The statistical test(s) used AND whether they are one- or two-sided<br>*Only common tests should be described solely by name; describe more complex techniques in the Methods section.*
☒	☐	A description of all covariates tested
☐	☒	A description of any assumptions or corrections, such as tests of normality and adjustment for multiple comparisons
☐	☒	A full description of the statistical parameters including central tendency (e.g. means) or other basic estimates (e.g. regression coefficient) AND variation (e.g. standard deviation) or associated estimates of uncertainty (e.g. confidence intervals)
☐	☒	For null hypothesis testing, the test statistic (e.g. *F*, *t*, *r*) with confidence intervals, effect sizes, degrees of freedom and *P* value noted<br>*Give P values as exact values whenever suitable.*
☒	☐	For Bayesian analysis, information on the choice of priors and Markov chain Monte Carlo settings
☒	☐	For hierarchical and complex designs, identification of the appropriate level for tests and full reporting of outcomes
☐	☒	Estimates of effect sizes (e.g. Cohen's *d*, Pearson's *r*), indicating how they were calculated

Our web collection on statistics for biologists contains articles on many of the points above.

Software and code

Policy information about availability of computer code

Data collection	The code used to query reference spectra of drugs is available on GitHub under the MSn library project (https://github.com/corinnabrungs/msn_tree_library). The code used to query FastMASST api in batch mode to search for drug analogs is available on GitHub (https://github.com/robinschmid/microbe_masst; accessed 03/2025). The code used to filter the drug analog matches is provided on GitHub (https://github.com/ninahaoqizhao/Manuscript_GNPS_Drug_Library) and Zenodo (https://doi.org/10.5281/zenodo.17232336).
Data analysis	The code used for dataset analysis can be found on GitHub (https://github.com/ninahaoqizhao/Manuscript_GNPS_Drug_Library and https://github.com/kinekvitne/manuscript_drug_library) and Zenodo (https://doi.org/10.5281/zenodo.17232336 and https://doi.org/10.5281/zenodo.17230320).

For manuscripts utilizing custom algorithms or software that are central to the research but not yet described in published literature, software must be made available to editors and reviewers. We strongly encourage code deposition in a community repository (e.g. GitHub). See the Nature Portfolio guidelines for submitting code & software for further information.

Data

Policy information about availability of data

All manuscripts must include a data availability statement. This statement should provide the following information, where applicable:

- Accession codes, unique identifiers, or web links for publicly available datasets
- A description of any restrictions on data availability
- For clinical datasets or third party data, please ensure that the statement adheres to our policy

The MGF spectral files for the GNPS Drug Library and the associated metadata of controlled vocabularies (.csv) have been deposited in Zenodo at https://doi.org/10.5281/zenodo.13892288. The downloaded MGF spectral files can be added to personal GNPS folders and used directly for library matching. Reference spectra of drugs in the GNPS Drug Library were extracted from the open access GNPS Spectral Library (accessed 08/2023). Drug analogs were mined from the MetaboLights, Metabolomics Workbench, and GNPS/MassIVE data repositories using fasstMASST api (https://fasst.gnps2.org/search; accessed 04/2025). The raw data files for the pharmacokinetics studies have been deposited in GNPS/MassIVE repository (https://massive.ucsd.edu; same below) under the accession numbers MSV000085944, MSV000084008, and MSV000082493. Raw data files from the American Gut Project have been deposited at MSV000080673. Raw data files for fecal samples from the HNRC cohort have been deposited at MSV000092833. Raw data files for the drug bacterial cultures have been deposited at MSV000095331. Raw data files for HNRC fecal samples analyzed with the bacterial cultures have been deposited at MSV000096012. Raw data files for co-migration of the bacterial cultures and fecal samples have been deposited at MSV000096013. Raw data files for wastewater influents have been deposited at MSV000097575. Due to human subject protection constraints, clinical metadata for the HNRC cohort will be provided upon request to HNRC: https://hnrp.hivresearch.ucsd.edu. Source data are provided with this paper.

Research involving human participants, their data, or biological material

Policy information about studies with human participants or human data. See also policy information about sex, gender (identity/presentation), and sexual orientation and race, ethnicity and racism.

Reporting on sex and gender	Drug profiles in different sex are compared using American Gut Project dataset. The term "sex" was used in the manuscript. The sex information was determined based on self-reporting. The information is not specifically collected for this study and is publicly available in the ReDU metadata (http://redu.ucsd.edu/) associated with the American Gut Project metabolomics dataset (MSV 000080673).
Reporting on race, ethnicity, or other socially relevant groupings	No race, ethnicity, or other socially relevant groupings are reported in the manuscript.
Population characteristics	The American Gut Project contains fecal samples from 1,993 subjects, with participants from the United States (US), Europe, and Australia with age 46 ± 18 years (range 3-93; 53% female). The HIV Neurobehavioral Research Center (HNRC) cohort provides 322 fecal samples for metabolomics analysis. Among them, 222 were from people with HIV and 100 were from people without HIV.
Recruitment	All subjects received oral and written information about the study according to Good Clinical Practice and written informed consent was obtained prior to any study specific procedures. More detailed information can be provided upon request. The American Gut Project (MSV000080673) and the two pharmacokinetic datasets, e.g. the diphenhydramine study (MSV000085944) and the Cooperstown cocktail study (MSV000084008 and MSV000082493) are public datasets. The HNRC data are not public due to human subject protection constraint.
Ethics oversight	All datasets included in the manuscript were approved by the Institutional Review Board for Human Research, either by the University of California San Diego or University of Colorado, Boulder, and performed in accordance with the Declaration of Helsinki: American Gut Project, protocol no. 141853 and 12-0582; diphenhydramine study, protocol no. 191026; Cooperstown cocktail study, protocol no. 161940; HNRC, 172092. All subjects provided written informed consent.

Note that full information on the approval of the study protocol must also be provided in the manuscript.

Field-specific reporting

Please select the one below that is the best fit for your research. If you are not sure, read the appropriate sections before making your selection.

☒ Life sciences ☐ Behavioural & social sciences ☐ Ecological, evolutionary & environmental sciences

For a reference copy of the document with all sections, see nature.com/documents/nr-reporting-summary-flat.pdf

Life sciences study design

All studies must disclose on these points even when the disclosure is negative.

Sample size	No statistical methods were used to predetermine sample sizes. Sample sizes were chosen based on common approaches in untargeted metabolomics studies and were constrained by the availability of biological material and resources. For microbial incubation experiments, three independent biological replicates per condition were performed, which is consistent with prior work in this field. For human cohort analyses, sample sizes were determined by the number of samples available.

Data exclusions	No data was excluded from the analysis.
Replication	For the drug microbial incubations, independent cultures were set up in triplicate for each drug mix for the two time points (n = 3 for each drug mix at each time point). Pairwise Wilcoxon tests were performed to compare the drug analog intensities in bacterial cultures at 0 hour and 72 hours. Due to the small sample size (n = 3 in each group), p-values <0.1 were considered as statistically significant.
Randomization	For LC–MS/MS analysis, all samples were randomized prior to instrument injection. Samples from human subjects were randomly plated into 96-well plates before extraction. For microbial incubations, three biological replicates per condition were placed together on the same plate to ensure consistent handling; however, the order of injection into the instrument was randomized across all samples.
Blinding	Blinding was not relevant to this study because we did not assign subjects to groups.

Reporting for specific materials, systems and methods

We require information from authors about some types of materials, experimental systems and methods used in many studies. Here, indicate whether each material, system or method listed is relevant to your study. If you are not sure if a list item applies to your research, read the appropriate section before selecting a response.

Materials & experimental systems

n/a	Involved in the study
☒	☐ Antibodies
☒	☐ Eukaryotic cell lines
☒	☐ Palaeontology and archaeology
☒	☐ Animals and other organisms
☒	☐ Clinical data
☒	☐ Dual use research of concern
☒	☐ Plants

Methods

n/a	Involved in the study
☒	☐ ChIP-seq
☒	☐ Flow cytometry
☒	☐ MRI-based neuroimaging

Plants

Seed stocks	Not used in this study.
Novel plant genotypes	Not used in this study.
Authentication	Not used in this study.
